# Human osteoclast formation and resorptive function on biomineralized collagen

**DOI:** 10.1016/j.bioactmat.2021.06.036

**Published:** 2021-07-15

**Authors:** Daniel de Melo Pereira, Noel Davison, Pamela Habibović

**Affiliations:** Department of Instructive Biomaterials Engineering, MERLN Institute for Technology-Inspired Regenerative Medicine, Maastricht University, 6229 ER, Maastricht, The Netherlands

**Keywords:** Osteoclastogenesis, Osteoclast resorption, Biomineralized collagen, Intrafibrillar mineral, Bone graft substitute

## Abstract

Biomineralized collagen composite materials pose an intriguing alternative to current synthetic bone graft substitutes by offering a biomimetic composition that closely resembles native bone. We hypothesize that this composite can undergo cellular resorption and remodeling similar to natural bone. We investigate the formation and activity of human osteoclasts cultured on biomineralized collagen and pure collagen membranes in comparison to cortical bone slices. Human monocytes/macrophages from peripheral blood differentiate into multinucleated, tartrate-resistant alkaline phosphatase (TRAP)-positive osteoclast-like cells on all substrates. These cells form clear actin rings on cortical bone, but not on biomineralized collagen or pure collagen membranes. Osteoclasts form resorption pits in cortical bone, resulting in higher calcium ion concentration in cell culture medium; however, osteoclast resorption of biomineralized collagen and collagen membranes does not measurably occur. Activity of osteoclast enzymes – TRAP, carbonic anhydrase II (CA-II), and cathepsin-K (CTS-K) – is similar on all substrates, despite phenotypic differences in actin ring formation and resorption. The mesh-like structure, relatively low stiffness, and lack of RGD-containing binding domains are likely the factors responsible for preventing formation of stable actin rings on and resorption of (biomineralized) collagen membranes. This insight helps to guide further research toward the optimized design of biomineralized collagen composites as a more biomimetic bone-graft substitute.

## Highlights

• Human osteoclasts differentiated on biomineralized collagen, but no resorption occured.
• Porous surface of fibrillary collagen and lack of RGD ligands to the vitronectin receptor likely prevented osteoclast resorption.
• Quantification of resorption on uneven surfaces in comparison with bone was possible with 3D laser microscopy.

## Graphical abstract

**Figure:**
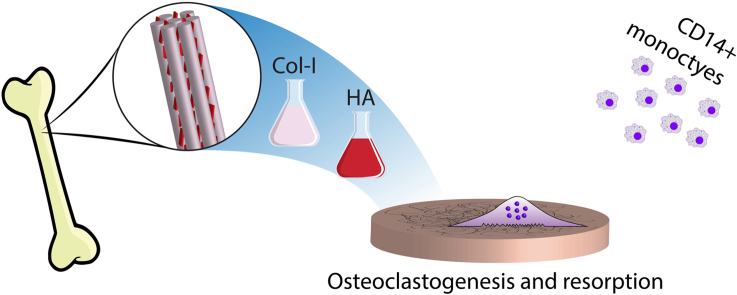

## 1 Introduction

Biomineralization is a process used by many organisms to integrate a mineral entity in an organic setting, with often fascinating results [1]. As the biomineralization process unfolds, there is tight spatial and temporal control over the availability of ions and mineral precursors, and over the way they organize with organic components. This allows for the creation of complex structures, in which function is directly linked to form. Excellent examples for this can be found in the human body, where our bones, teeth, and inner ear are biomineralized tissues with unique properties and function [2]. One of the key functions of human skeleton is to provide mechanical support and protection to the soft tissues and organs, for which exceptional mechanical properties are a prerequisite. Bone possesses a unique combination of stiffness and fracture toughness that cannot be solely explained by its composition. In fact, the structural arrangement between soft (organic) and hard (inorganic) components is critical to its performance [3,4]. Biomineralized tissues and the processes by which they form are therefore a natural source of inspiration for the development of new and improved biomaterials.

Many currently available synthetic substitutes of natural bone grafts (auto-, allo- and xenografts) are comprised of collagen, various types of calcium phosphates (CaP), or combinations thereof, inspired by the composition of natural bone tissue [5,6]. Although such biomaterials (partially) mimic the composition of bone, their structure normally does not resemble the intricate organic-inorganic architecture of bone at the submicron scale. As a result, their mechanical properties typically fall short of what is clinically required, especially in load-bearing applications. Moreover, the microenvironment in which the cells relevant to bone formation and remodeling reside is different from their natural microenvironment.

These two drawbacks of current synthetic bone grafts are addressed in biomineralized collagen, consisting of collagen with intrafibrillar CaP mineral, a material that can be prepared in the laboratory, owing to advances in understanding bone biomineralization processes [7,8]. It is therefore important to further expand our knowledge of biomimetic materials based on biomineralized collagen, and use this knowledge to develop improved bone graft substitutes.

Intrafibrillarly mineralized collagen is commonly prepared by a method named polymer-induced liquid precursor (PILP) method, in which an amorphous calcium phosphate (ACP) phase, stabilized by charged polymers (e.g. poly-l-aspartic acid or poly-l-glutamic acid) [9], is able to migrate to the inner confined spaces of collagen-I fibers, where it crystallizes into nano-sized hydroxyapatite (HA) platelets, resulting in intrafibrillary mineralized fibers [[10], [11], [12]]. Biomimetic materials based on intrafibrillarly-mineralized collagen replicate key aspects of bone composition and structure to a degree that was not possible before, and are therefore interesting to investigate in the context of bone regeneration. Of note, at the individual fibril level, biomineralized collagen has mineral density similar to bone, as well as similar organization of its main organic and inorganic components, with apatite platelets arranged with their c-axis quasi-parallel to the longitudinal direction of the collagen fiber. The parallel organization of stiffer elongated apatite particles inside the softer collagen matrix phase is likely to contribute to the mechanical properties exhibited by bone at a larger scale [13,14]. Therefore, this mineralized fibril motif constitutes, essentially, the building block of bone's extracellular matrix (ECM), being therefore a promising candidate for building larger scaffolds/constructs that can eventually combine bone-like mechanical properties and bioactivity.

One of the key properties of graft substitute materials for regeneration of large bone defects is their rate of degradation and replacement by native tissue. CaP-based materials, commonly used as synthetic bone graft substitutes, when implanted in a bone environment undergo degradation by both passive physicochemical processes such as hydrolytic dissolution and (mechanical) fragmentation, and by cell-mediated processes such as phagocytosis by macrophages and resorption by osteoclasts [15]. Cell-mediated processes are particularly interesting from the perspective of designing materials that are capable of responding to and interacting with the implant environment. Resorption of old ECM by osteoclasts and deposition of new bone tissue by osteoblasts are the two continuous processes responsible for maintaining bone homeostasis. Coordination between these two tightly synchronized processes is ensured by cross-talk between osteoclasts and osteoblasts in a process called bone coupling [16]. When living bone tissue is used as a bone graft material, the graft is slowly resorbed and gradually replaced by new bone, beginning from the edges of the defect toward the middle. This process is defined as creeping substitution of the graft and importantly results in little mechanical weakening of the graft prior to complete bone repair [17]. From this perspective, a synthetic graft material that is recognized and resorbed by resident osteoclasts may elicit similar crosstalk and osteogenic coupling factors [18], thereby retaining its mechanical properties as it is selectively substituted by new bone.

Toward the aim of incorporating bone graft substitutes into the bone remodeling system, it is important to investigate whether biomineralized collagen can undergo resorption by osteoclasts, as this is a first step for cell-mediated synchronous removal of the bone graft substitute and replacement by native tissue. If osteoclast-mediated creeping substitution of a biomimetic bone graft substitute could be achieved, it would potentially avoid the challenges in engineering responsive physicochemical degradation rates to match dynamically variable bone formation.

To our knowledge, so far, no studies have directly evaluated osteoclast formation and function on synthetic biomineralized collagen matrices, i.e., collagen matrices with intrafibrillar mineral, although other collagen-hydroxyapatite scaffolds have been investigated in this context [19,20]. Indirect approaches have however been used. Mouse osteoclasts were shown to resorb demineralized bone that was re-mineralized by the PILP method, with osteopontin as process-directing agent instead of a polymer [21]. Mouse mesenchymal stromal cells (MSCs), cultured in a *trans*-well system in the presence of collagen scaffolds containing intrafibrillar apatite and silica, were shown to have an inhibitory effect on osteoclastogenesis and resorption when subsequently co-cultured with mouse macrophage cell line RAW 264.7, showcasing the type of cell-to-cell communication that can be affected by these biomaterials [22].

To expand the current knowledge on cell-mediated degradation of biomineralized collagen, we differentiated human monocytes into osteoclasts on mineralized collagen substrates produced with the PILP method and evaluated osteoclast formation and function. For comparison, collagen matrices without mineral, as well as cortical bone slices, a well-known control surface for in-vitro osteoclast resorption studies, were used.

## 2 Materials and methods

### 2.1 Materials

PureCol® collagen type-I solution (col-I, 3 mg/mL, 97% bovine dermal type I atelo-collagen) was purchased from Advanced BioMatrix (San Diego, California, USA, cat#5005). Poly-l-aspartic acid sodium salt (pAsp, Mw = 27 kDa) was purchased from Alamanda Polymers (Huntsville, Alabama, USA, cat#000-D200). 1-Ethyl-3-[3-dimethylaminopropyl] carbodiimide hydrochloride (EDC), N-hydroxysulfosuccinimide (sulfo-NHS), calcium chloride dihydrate, potassium phosphate dibasic, and all other chemicals were ordered from Sigma-Aldrich (St. Louis, Missouri, USA). Recombinant human M-CSF and soluble RANK-L cytokines were from Peprotech (Connecticut, USA, cat#3002510 and cat#3100110).

### 2.2 Preparation of collagen membranes

In brief, collagen gels with a concentration of 2 mg/mL were prepared by mixing 1412 μL of collagen solution, 352 μL of 10*x* phosphate buffered saline (PBS), and 236 μL of 0.1 M NaOH. Gels with a volume of 2 mL were formed in modified 5 mL syringes (Sigma Aldrich, cat#Z248010), at 37 °C for 24 h. The syringes containing the gel were inverted on top of nylon meshes with a pore size of 40 μm (Fischer Scientific, cat #11587522) and left for 24 h at 37 °C to allow the gels to loose water under their own weight. After this compression step, syringes were removed, and the formed collagen membranes remained in the cell strainer for another 24 h, before being washed with PBS and cross-linked with 50 mM EDC, 25 mM sulfo-NHS in 50 mM MES buffer (pH = 7.0), overnight at room temperature. The procedure for making collagen membranes is illustrated in Supplementary Fig. 1. The following day, the membranes were washed with PBS, incubated with 0.1 Na_2_HPO_4_ and 2 M NaCl for 2 h, to quench the remaining activated carboxylic acid residues, and finally washed three times with PBS for 2 h. The membranes were kept in PBS at 4 °C for up to one week before being used for cell culture or characterization. All steps were performed in a sterile environment.

### 2.3 Mineralization of collagen membranes

Mineralization solution was prepared according to the PILP method [8]. Briefly, precursor solutions of calcium (9 mM CaCl_2_) and phosphate (4.2 mM K_2_HPO_4_) were prepared in a buffer with 50 mM TRIS base and 150 NaCl keeping pH at 7.8–8.0 at room temperature. Prior to incubation, pAsp was added to the calcium precursor solution, mixed and allowed to rest for 5 min, followed by addition of the same volume of phosphate precursor solution. The final concentration of pAsp was 100 μg/mL. After mixing the two precursor solutions, the collagen membranes were added (40 mL of solution was used per membrane) and left at 37 °C for 7 days. Before cell culture, mineralized membranes were washed with PBS and incubated in cell culture medium for 3–4 h. All steps were performed in a sterile environment.

### 2.4 Characterization of collagen and biomineralized collagen membranes

#### 2.4.1 Scanning electron microscopy and energy-dispersive X-ray spectroscopy (SEM-EDS)

For SEM-EDS, collagen and biomineralized collagen membranes were dehydrated in a series of ethanol in PBS (30, 40, 50%), followed by a series of ethanol in water (60, 70, 80, 90, 100%), 15 min per step and finally in hexadimethylsiloxane for 30 min before air drying overnight. Samples were glued with silver paint (Ted Pella, USA, cat#16062) onto aluminum stubs and sputter-coated with a 2 nm layer of iridium for SEM imaging, or left without coating for elemental analysis. Imaging was done at 2–10 kV at a working distance of 2–10 mm with a Teneo microscope (FEI, USA) using the T1 or ETD detectors in Optiplan mode. Elemental analysis was done by EDS (Team EDS from EDAX, USA) on a VERSA electron microscope (FEI, USA).

#### 2.4.2 Transmission electron microscopy and selected area electron diffraction (TEM-SAED)

For TEM-SAED, collagen fibrils were formed on a gold grid (Electron Microscope Sciences, USA, cat#FCF400–Au) by placing the grid on a 50 μL droplet of col-I solution (2 mg/mL as described above before the compression step) and incubating at 37 °C in a humid environment. After washing with distilled water, the collagen-coated grid was placed on top of a 50 μL droplet of mineralization solution (prepared as described above) and incubated for 1 or 7 days at 37 °C. Finally the grids were washed with distilled water and air-dried at room temperature overnight. Samples were imaged at 120 kV (Tecnai G2 Spirit, FEI, OR, United Stated). For calculating the d-spacings from electron diffraction, a new gold grid was used to obtain the diffraction pattern of gold which was compared with known d-spacings, from which the reciprocal space scale was calculated.

#### 2.4.3 Thermogravimetric analysis (TGA)

For TGA, collagen and biomineralized collagen membranes were washed with MilliQ water to remove salts, blotted with filter paper and stored in microcentrifuge tubes. Samples were burned in air, up to 800 °C, at a rate of 5 °C/min, using a Q500 TGA (TA Instruments, Belgium). The leftover weight at 700 °C was considered to be the mineral content of the membrane [23].

### 2.5 Monocyte/macrophage culture and differentiation into osteoclasts

Poietics^tm^ human peripheral blood CD14^+^ monocytes were obtained from Lonza (Basel, Switzerland, cat#2W–400B, lot#647890). Upon thawing the cryovial, cells were transferred to a 15 mL tube and 9 mL warm basic cell culture medium (α-MEM (Lonza, cat#BE02-002F) supplemented with 10% (v/v) HyClone^tm^ FetalClone^tm^ serum (Thermo Fisher Scientific, USA, cat#10780245, lot#HXSH3008003), 100 U/ml penicillin, 100 μg/mL streptomycin and 20 ng/mL M-CSF) was added, followed by centrifugation at 300 rcf for 5 min. Cells were re-suspended in basic cell culture medium, counted, and seeded at a density of 500.000 cells/cm^2^ into 96-well or 48-well plates containing the various substrates. For the first 3 days of culture, the basic medium was modified to contain 35 ng/mL M-CSF. Then, the culture continued in either basic (20 ng/mL M-CSF) or in differentiation medium (basic medium supplemented with 40 ng/mL RANK-L) for up to 21 days, counting from the addition of RANK-L, with medium refreshment every 2–3 days.

### 2.6 Immunohistochemical analysis of osteoclasts by confocal laser microscopy

After 14 or 21 days of culture, cells on different substrates were washed once with warm PBS, and fixed with warm 10% formaldehyde in PBS for 15 min at room temperature. Cells were washed three times with PBS, and permeabilized with 0.2% (v/v) Tween-20 in PBS for 10 min at room temperature. ELF-97 phosphate substrate (Thermo Fisher Scientific, USA, cat#E6601) was used for visualization of activity of tartrate-resistant alkaline phosphatase (TRAP) according to the protocol published by Filgueira [24]. Briefly, 20*x* concentrated ELF-97 substrate was diluted in a buffer composed of 110 mM acetate (pH = 5.2), 1.1 mM sodium nitrite and 7.4 mM tartrate. Cells were incubated with diluted ELF-97 for 15 min, washed three times with PBS, incubated with 1:200 PBS-diluted AlexaFluor® 488 phalloidin (Thermo Fisher Scientific)) for 20 min, washed three times with PBS and finally incubated with 1 μg/mL DAPI (Thermo Fisher Scientific, USA, cat#1306) in PBS for 10 min. All incubation steps were at room temperature, in the dark. Imaging (n = 2) was done with a SP8 STED confocal laser microscope (Leica Microsystems, Germany) using 25*x* and 86*x* water-immersion objectives. Sequential scanning was performed with two detector configurations, one for DAPI and ELF-97, and another for phalloidin 488. This allowed observation of ELF-97 and phalloidin AF-488 signals without any signal interference, despite the considerable overlap of the emission wavelength of both fluorophores. A 405 nm excitation laser was used for DAPI and ELF-97, with detection wavelengths of 417–478 nm for DAPI and 570–621 nm for ELF-97. A 488 nm excitation laser was used for phalloidin AF-488, with detection wavelengths of 499–570 nm. Large, multinucleated (n > 2) and TRAP-positive cells were considered to be osteoclasts. Quantification of TRAP activity was performed by image analysis using Image J (version 1.52r). The number of TRAP positive pixels was counted for each osteoclast, and expressed as fraction of total cell area. Details of this analysis are provided in a short video as a part of the available dataset published with this study.

### 2.7 Identification and quantification of resorption sites

#### 2.7.1 Optical profilometry

After 21 days, cells were removed from the substrates by incubation in 1% Triton X-100 in PBS for 30 min in an ultrasonic waterbath with ice. The substrates were washed with distilled water and air-dried before analysis. Bone slices, collagen and biomineralized collagen membranes (n = 3) were imaged with a VKX-200 3D laser microscope (Keyence, Japan) using the 20*x* objective. All substrates were imaged completely by stitching. A reference surface (z = 0) was defined for individual images using a planar tilt correction (bone slice) or a waveform removal with cutoff set at 200 μm ((biomineralized) collagen membranes). A threshold below the reference surface was set for bone slices (z = −2 μm) and for both biomimetic membranes (z = −5 μm), and all data points below this threshold were considered a result of resorption. Quantification was done using the volumetric tool.

#### 2.7.2 SEM

After 21 days, the cells were either fixed on or removed from the substrates, as described above. The substrates with cells were dehydrated in a series of ethanol in PBS (30, 40, 50%), followed by a series of ethanol in water (60, 70, 80, 90, 100%), 15 min per step and finally in hexadimethylsiloxane for 30 min before air drying overnight. Samples were glued onto aluminium stubs with carbon tape and silver paint, and sputter-coated with a 2 nm layer of iridium. Imaging was done in a TENEO electron microscope, using the T1 detector in Optiplan mode, with the beam at 2–10 kV and working distance of 2–10 mm.

#### 2.7.3 Ion-coupled plasma mass spectrometry (ICP-MS)

At each medium refreshment, a 100 μL-sample of medium was collected and kept at −30 °C until analysis. After collecting samples at the different time points, they were thawed, and 50 μL of cell culture medium was diluted in 950 μL measuring matrix (1% HNO_3_ with 20 ppb scandium). A standard curve of calcium and phosphorous was prepared, ranging from 100 to 4000 ppb. Scandium was used as internal standard to compensate signal drift from the instrument and other factors. For each type of substrate analyzed (bone slice and biomineralized collagen), there were two conditions (basic medium and differentiation medium) each with 3 replicates, i.e. medium collected from 3 different wells of a well plate. Since collagen membranes without mineral did not contain any calcium, the ICP-MS analysis for this condition was not performed.

### 2.8 Enzymatic activity assays

At 7, 14 or 21 days, the cell culture medium was removed and substrates were washed once with warm PBS. PBS was removed before freezing the plates containing samples at −80 °C. When the samples at different time points were collected, the plates were thawed, the substrates were transferred into new plates, and 200 μL lysis buffer (1% Triton X-100 in PBS) was added to each well. Two more freeze-thaw cycles were performed, followed by a 30-min incubation in an ultrasonic waterbath with ice. Cell lysates were then transferred to 0.5 mL microcentrifuge tubes and either analyzed immediately, or frozen for later analysis. All handling of cell lysate samples was performed in ice to delay protein degradation.

#### 2.8.1 Quantification of tartrate-resistant alkaline phosphatase (TRAP) activity

For quantification of TRAP activity (n = 3), 20 μL of cell lysate was mixed with 130 μL of buffer containing 10 mM 4-nitrophenylphosphate, 0.1 M sodium acetate (pH 5.8), 0.15 M KCl, 0.1% Triton X-100, 10 mM sodium tartrate, 1 mM ascorbic acid, and 0.1 mM FeCl6. The mixture was incubated at 37 °C for 1 h, quenched with 100 μL 0.3 M NaOH, and finally the absorption was measured at 405 nm. A standard curve of 4-nitrophenol was used to convert absorption values into moles of liberated phosphate.

#### 2.8.2 Carbonic anhydrase II (CA-II) activity

Quantification of CA-II activity (n = 3) was done by proxy via its known esterase activity. In brief, 50 μL of cell lysate was added to 50 μL of buffer, containing 12.5 mM Tris (pH = 7.5), 75 mM NaCl and 2 mM 4-nitrophenylacetate. Absorbance was measured after 5 min at 405 nm with a CLARIOstar Plus microplate reader (BGM Labtech, Germany). A standard curve of 4-nitrophenol was used to convert absorption values into moles of liberated acetate.

#### 2.8.3 Quantification of cathepsin-K (CTS-K) activity

Quantification of CTS-K activity (n = 3) was performed as described before [25]. Briefly, 10 μL of cell lysate was further diluted in 40 μL of PBS, and then mixed with 50 μL buffer containing 0.1 M sodium acetate, 4 mM EDTA, 4 mM DTT (pH = 5.5) and 100 μM Z-LR-AMC. The plate was incubated at 37 °C for 30 min, and fluorescence was measured at an excitation/emission wavelength of 365/440 nm. A standard curve of aminomethylcoumarin (AMC) was used to convert the fluorescence intensity into moles of liberated AMC.

### 2.9 Statistical analysis

Statistical analysis was performed in GraphPad Prism (version 8.3). The Student's t-test was used to compare resorption data from optical profilometry between cells cultured in basic and differentiation medium for each substrate, and two-way ANOVA was used to compare Ca^2+^ data from ICP-MS as well as enzymatic activity data from the respective assays. Differences in means between conditions were tested at each timepoint. A Bonferroni post-hoc test was used to correct for multiple comparisons (one family for all comparisons). Mean differences were considered statistically significant for p-value < 0.05. In all figures, the following notation applies: *p < 0.05; **p < 0.001; ***p < 0.0001.

## 3 Results and discussion

In this study, we aimed to investigate the behavior of human osteoclasts on synthetic biomineralized collagen in comparison to pure collagen and bovine bone slices. Understanding osteoclast formation, function, and related cell-mediated degradation of biomaterials is important for developing biomimetic bone graft substitutes based on biomineralized collagen.

### 3.1 Biomineralized membranes mimic composition and structure of bone at submicron scale

**Fig. 1:**
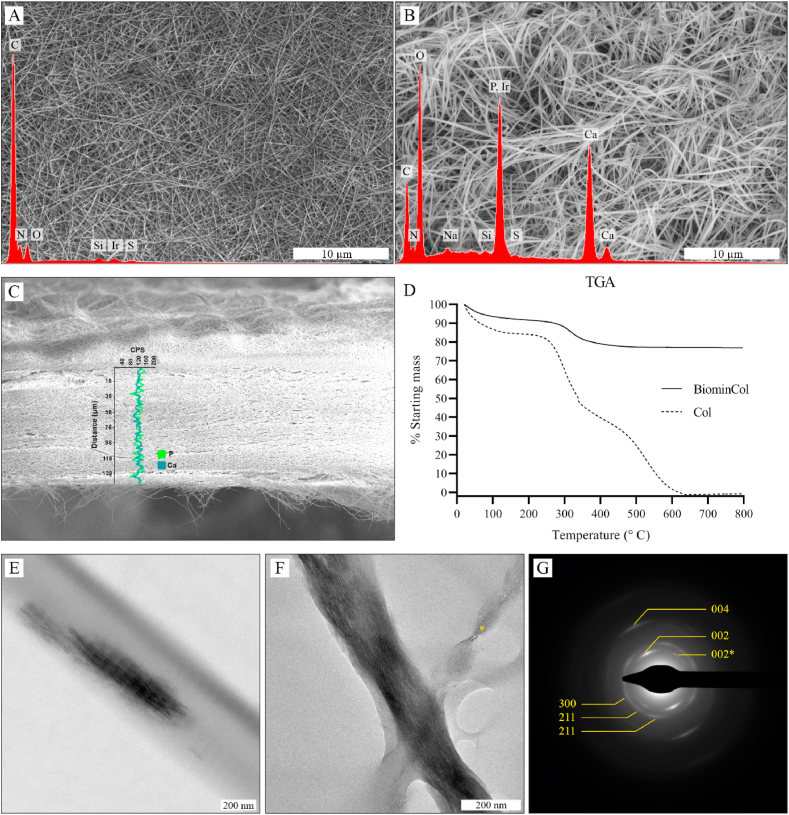
Characterization of collagen and biomineralized collagen membranes. SEM and corresponding EDS of a collagen membrane (A) and biomineralized collagen membrane (B). Cross-section SEM micrograph of a biomineralized collagen membrane, and EDS line scan analysis over its thickness (calcium in blue, phosphorus in green) (C). Quantification of mineral content by TGA of collagen (Col) and biomineralized collagen (BiominCol) (D). TEM micrographs of collagen fibrils after 1 day (E) and 7 days (F) of mineralization using the PILP method. SAED of the area imaged in (F), with the diffraction arcs corresponding to hydroxyapatite reflections highlighted (G). The 002* reflection corresponds to the fibril marked with * in (F).

Bovine collagen membranes were successfully produced and used as a base material in this study. The membranes had a porous, mesh-like structure, composed of randomly oriented collagen fibers, as shown by SEM (Fig. 1A). After mineralization using the PILP method, no nodular mineral deposits were observed on the collagen fiber surface, as is the case with conventional CaP coating methods, such as incubation in simulated body fluid (SBF) [26]. Fiber diameter increased from 81 ± 23 to 185 ± 38 (n = 20) after the mineralization process and elemental analysis by EDS showed presence of calcium and phosphorus (Fig. 1B) which was not the case when pure collagen was analyzed (Fig. 1A). An EDS line scan over the cross section of a biomineralized membrane detected calcium and phosphorus across the entire thickness of the membrane (about 100 μm) and not only on the surface, indicating that the polymer-ACP complexes were able to diffuse through the membrane and mineralize it completely (Fig. 1C). TGA indicated that the inorganic mass fraction (intrafibrillar CaP mineral) was about 78%, while collagen and residual water mass fraction made up the remaining 22% that were burned away after heating to 800 °C (Fig. 1D). These results provided convincing evidence that CaP mineral was present in the biomineralized membrane, in amounts similar to that of secondary bone, consisting of about 65 wt% mineral phase, 25 wt% organic (of which around 90% is collagen type I), and 10 wt% water [7], and that its distribution throughout the membrane was homogeneous. Moreover, as mineral was not observed on the surface of collagen fibers, it likely deposited within the fibers as intrafibrillar mineral. Further confirmation was obtained by TEM, where, after incubation of collagen fibers for 1 day in the PILP mineralization solution, nano-sized crystal particles were observed in close association with collagen fibers, as dark streaks parallel to each other and aligned with the collagen fiber direction (Fig. 1E). Evidence of the identity of these mineral deposits was obtained after 7 days of mineralization by SAED of a fully mineralized collagen fiber, which showed diffraction arcs with d-spacings and angles corresponding to reflections from HA crystal planes 002, 004, 112, 211 and 300 (Fig. 1F and G) [7]. The 002 and 004 planes are normal to the c-axis of the hexagonal crystal cell, and the narrow angle of their arcs in the diffraction pattern corresponds to the angular dispersion of the c-axis direction of HA crystal platelets, showing that the c-axis is aligned with the direction of the collagen fiber.

These results show that CaP mineral was present in the form of nano-sized HA crystals embedded in the collagen fibers, making this biomimetic material similar to bone in composition and structure, at the length scale of nano-to micrometer [8].

### 3.2 Multinucleated osteoclast-like cells are able to form on collagen, biomineralized collagen as well as on cortical bone slices

**Fig. 2:**
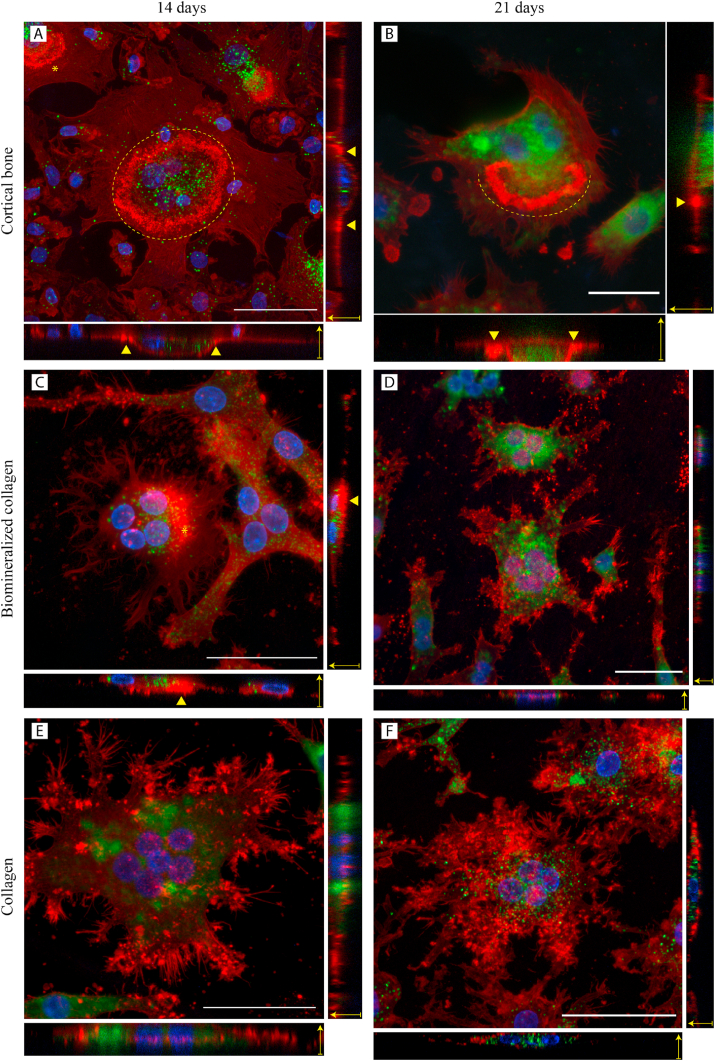
Osteoclasts on cortical bone, biomineralized collagen and collagen surfaces after 14 or 21 days of culture in differentiation medium. F-actin (red), nuclei (blue) and TRAP (green). In cortical bone (A,B), bright actin staining shows organized actin ring structures, crescent shaped (pointed in A by *) or circular (traced line in A), are visible within the cell. Orthogonal projections of xz (bottom) and yz (right side) show that these structures (arrowheads) are enclosing a basal recess oriented deeper into the substrate surface than the adjacent substrate surface, suggesting these are sealing zones undergoing active resorption. Arrows in the orthogonal projections indicate the vertical direction. Actin rings are still visible in cortical bone after 21 days (traced line in B). Brightly stained punctate actin structures (*) are also seen in some osteoclasts on biomineralized collagen (C,D), resembling podosomes but without the ring-like organization seen in cortical bone. Side view shows that these structures (arrowheads) are likely in contact with the surface of the material, but they do not as clearly enclose a contiguous zone as observed in cortical bone. Similar structures appeared less often and not as concentrated on collagen surfaces (E,F). All scale bars are 50 μm.

Upon optimization of culture protocols, it was possible to differentiate human peripheral blood-derived monocytes into osteoclast-like cells when cultured on the biomineralized collagen membranes, as well as on collagen membranes without mineral and cortical bone slices. Large, multinucleated, and TRAP-positive cells were observed on all three surfaces after 14 days of culture in differentiation medium (Fig. 2A, 2C, 2E). On cortical bone slices, F-actin structures having high fluorescence intensity were observed in the form of ring and crescent shapes with punctate morphology (Fig. 2A). These structures were different from other actin structures which were also observed, such as stress fibers. The high intensity punctate structures represent podosomal belts and actin rings, which define the sealing zone and resorption lacunae [27]. Actin rings provide evidence that osteoclasts are functional, able to seal off a compartment of the underlying material for resorption. Actin structures with high fluorescence intensity, likely podosomes, were also observed in osteoclasts in close contact with both collagen and biomineralized collagen membranes, but they lacked the organization seen on cortical bone slices, not having a clear dotted pattern or a well-defined ring- or crescent shape (Fig. 2C and E).

**Fig. 3:**
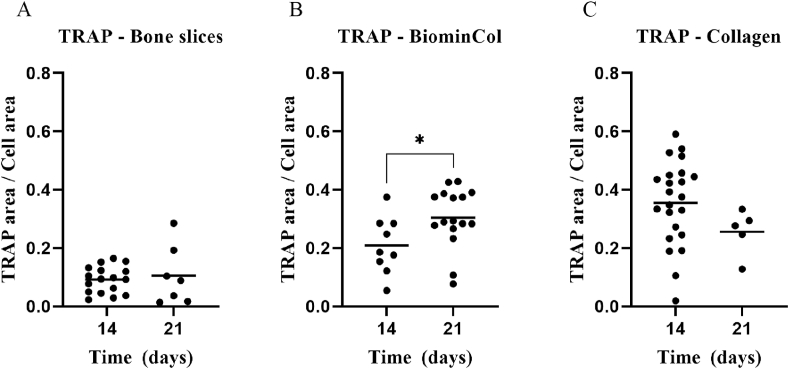
Semi-quantitative comparison of TRAP activity between osteoclasts cultured for 14 or 21 days on cortical bone slices, biomineralized collagen and collagen membranes. TRAP activity is shown as the area fraction of TRAP-positive pixels relative to the whole cell area, for each osteoclast (each point represents one osteoclast).

Similar observations were made after 21 days of culture (Fig. 2B, 2D, 2F). Actin rings were visible on cortical bone slices, indicating that osteoclasts were still active at this time point (Fig. 2B). Actin structures observed on the ventral membrane of cells on collagen membranes with and without intrafibrillar mineral did not display the structural organization of podosome belts or actin rings, even after 21 days (Fig. 2D and 2F). Quantification of TRAP activity by image analysis after 14 and 21 days of culture showed similar levels of activity per osteoclast on cortical bone slices, biomineralized collagen and collagen without mineral (Fig. 3).

These results suggest that, while osteoclast-like cells have the ability to form on collagen and biomineralized collagen surfaces, their function is likely to be impaired, given that the typical structures associated with functional osteoclasts were not observed after 14 or 21 days of culture in differentiation medium. This seems to be a direct effect of the properties of these biomimetic membranes, as mature, resorbing osteoclasts were able to form on the cortical bone slices under the same conditions.

It is generally recognized that both the physical and chemical properties of the substrate have an effect on osteoclast formation, surface adhesion and normal function, i.e. resorptive activity. Presence of integrin-binding motifs [28,29], substrate stiffness [30,31], porosity [32], and topographical roughness [27], were all shown to affect actin ring formation or resorptive activity.

Of particular interest to this study are the effects of integrin-binding motifs and physical properties of the biomineralized collagen membranes and collagen membranes without mineral, as these are the two major properties that distinguish these biomimetic materials from cortical bone. Cell-recognized, integrin-binding RGD sequences are present in various proteins of the ECM of cortical bone [33], but not in (biomineralized) collagen membranes [34,35]. The RGD motif is important for osteoclast adhesion and activation because it is the ligand to integrin α_v_β_3_, also known as vitronectin receptor, the most highly expressed integrin in osteoclasts, for which there is ample evidence that, in its absence, no resorption takes place [36,37]. However, even in the absence of RGD, osteoclasts have shown the ability to form actin rings on collagen-based surfaces, given the right physical properties. In the study by Nakamura et al., it was shown that osteoclasts were able to form actin rings when cultured on collagen-coated plastic coverslips, but not when residing in a collagen gel [29]. Importantly, the collagen-coated substrates were likely stiffer, flatter and less porous than the collagen gels, which were softer and presented a fibrillary, mesh-like surface topography. These differences plausibly impacted osteoclast function. Based on these findings, the authors suggested that actin ring formation depends not only on RGD binding but also on the physical properties of the substrate such as stiffness and surface topography. In comparison, the mesh-like fibrillary structure of the collagen membranes used in the present study was more similar to the collagen gel than to the collagen-coated coverslips described by Nakamura et al., in terms of surface structure and mechanical properties [47], which may explain why no resorption lacunae were observed on these surfaces. While it is difficult to determine precisely which material property was most consequential for this cellular effect, Touaitahuata et al. [31] showed that osteoclasts formed actin rings on softer materials like polyacrylamide gels and polydimethylsiloxane, with stiffness ranging from 30 to around 1800 kPa, suggesting that the porous surface structure rather than stiffness may be the dominant causal factor. Porosity, and particularly pore size, is known to impact osteoclast function, as shown by Davison et al. in a study with β-TCP ceramic discs with differently sized surface microstructures (grains and pores). No actin rings were observed for osteoclasts on β-TCP surfaces with micron-sized structural features (grain diameter 3.7 μm and pore size 1.8 μm), and there was also no resorption, but on β-TCP surfaces with submicron features (grain diameter 0.9 μm and pore size 0.6 μm) osteoclasts showed clear actin rings and there was ample resorption. This is in line with findings about the formation and stability of sealing zones on titanium surfaces of varying roughness, where an inverse correlation was found between the stability and translocation rate of sealing zones and roughness increasing from R_a_ = 1–4.5 μm [27]. This data suggests an interplay between stiffness, pore size and surface structure, which in our study was such that it inhibited actin ring formation and resorption.

### 3.3 Osteoclasts are able to resorb cortical bone but they do not resorb collagen and biomineralized collagen membranes

**Fig. 4:**
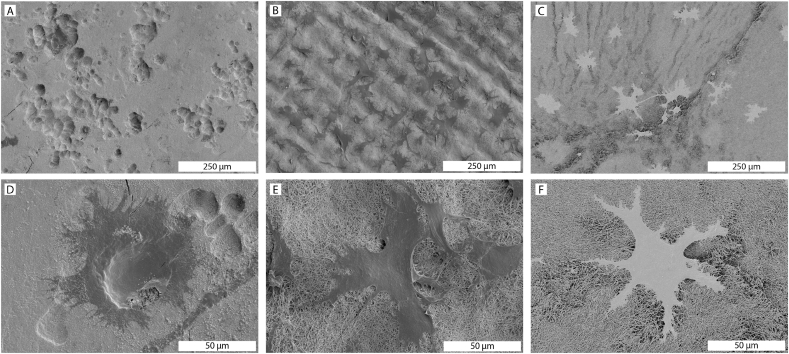
SEM images of osteoclasts on cortical bone (A,D), biomineralized collagen (B,E) and collagen (C,F) surfaces after 21 days in culture. Resorption lacunae can be seen in cortical bone, but not on biomineralized collagen or on collagen membranes.

Following an osteoclast culture of 21 days in differentiation medium, SEM analysis of the different surfaces was performed. Abundant resorption pits were observed on cortical bone slices (Fig. 4A and 4D). In contrast, no evidence for resorption on either biomineralized collagen membranes or collagen membranes without mineral was found (Fig. 4B, 4E, 4C, 4F). Occasionally, the fibrillar structure of the membrane surface in close contact with osteoclasts appeared to be disturbed and disorganized compared to the surrounding, cell-free surface, but it is unclear whether this is an effect of resorption or other cell-mediated effects, such as tension exerted on the fibers or enzymatic activity.

**Fig. 5:**
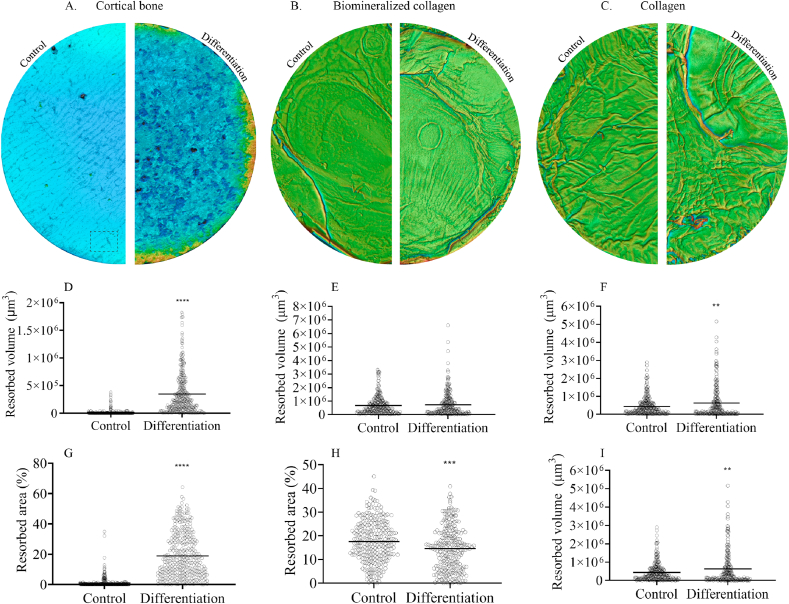
Volume and area resorbed by osteoclasts cultured on cortical bone, biomineralized collagen and collagen surfaces. The control condition were cells cultured in basic medium, with 20 ng/mL M-CSF but without RANK-L, whereas the differentiation medium contained 20 ng/mL M-CSF and 40 ng/mL RANK-L. Each data point represents a field-of-view as captured and analyzed by optical profilometry (dashed rectangle). Differences in resorbed volume and area between control and differentiation conditions are significant for cortical bone (p < 0.0001). Difference in resorbed area is significant for biomineralized collagen (p < 0.001). Difference in resorbed volume is significant for collagen (p = 0.0034).

Images of the surface made using an optical profilometer and the corresponding image quantification corroborated that extensive resorption occurred on cortical bone slices (Fig. 5A, 5D, 5G). Bone slices on which cells were cultured in the differentiation medium showed a mean resorbed volume that was about 25 times higher than when cells were cultured in basic medium, without RANK-L (Fig. 5D). Similarly, the resorbed area, expressed as a percentage of total area, was 1% and 19% when cells were cultured in basic and differentiation medium, respectively (Fig. 5G). Both difference in resorbed volume and area were statistically significant, with p < 0.0001.

While optical profilometry is a useful tool for analyzing the extent of resorption on bone slices, which are relatively smooth, this type of analysis is more difficult to perform on rough and uneven surfaces, because the algorithm is not able to differentiate between native topographical features (e.g. collagen fibers) and resorbed pits or trenches. As a result, more noise is present in the data, obscuring small differences in potential resorption; because of this, only very extensive resorption activity such as what was seen on cortical bone is robustly detectable. With this limitation of the technique in mind, the results showed that no significant difference in the mean resorbed volume was observed between cells cultured on biomineralized collagen membranes in basic or differentiation medium (Fig. 5E). A difference of 3% (p < 0.001) was observed for the resorbed area, with a larger area for cells cultured in basic medium, which is plausibly an artifact of the technique used (Fig. 5H). Similar to the results obtained for mineralized collagen membranes, cells cultured on collagen without mineral in differentiation medium showed a slightly higher mean resorbed volume (1.4 times (p = 0.0034)) than the control (Fig. 5F), while no significant differences were found in the percentage resorbed area (Fig. 5I). Notably, the percentage “resorbed area” in both basic and differentiation medium for both pure collagen and mineralized collagen was almost as high as the value observed for cells cultured on bone slices in the differentiation medium, which clearly shows that on these porous surfaces, the technique cannot distinguish between the structural properties of the material itself and structural changes resulting from resorption.

To make the technique suitable for analysis of rough surfaces, it is important to have a proper definition of a reference plane or level (z = 0), from which any concavity (with z < 0), would be counted as resulting from cell-induced resorption. The reference value for a relatively flat surface with low roughness (R_a_ < 1 μm), such as the bone slice, is easy to define with good accuracy, for example by definition of a reference plane. A surface presenting surface topography such as wrinkles or complex shapes in the tens or hundreds of micrometers, requires a more complex method for defining a good reference, for example using a band-pass filter that removes (i.e. makes z = 0) any feature larger than a pre-defined cutoff wavelength. The trade off in choosing the cutoff dimension involves over-estimation of resorption (cutoff dimension too large) or under-estimation of resorption (cutoff dimension too small), which translates as more noise across all conditions, as noted above.

**Fig. 6:**
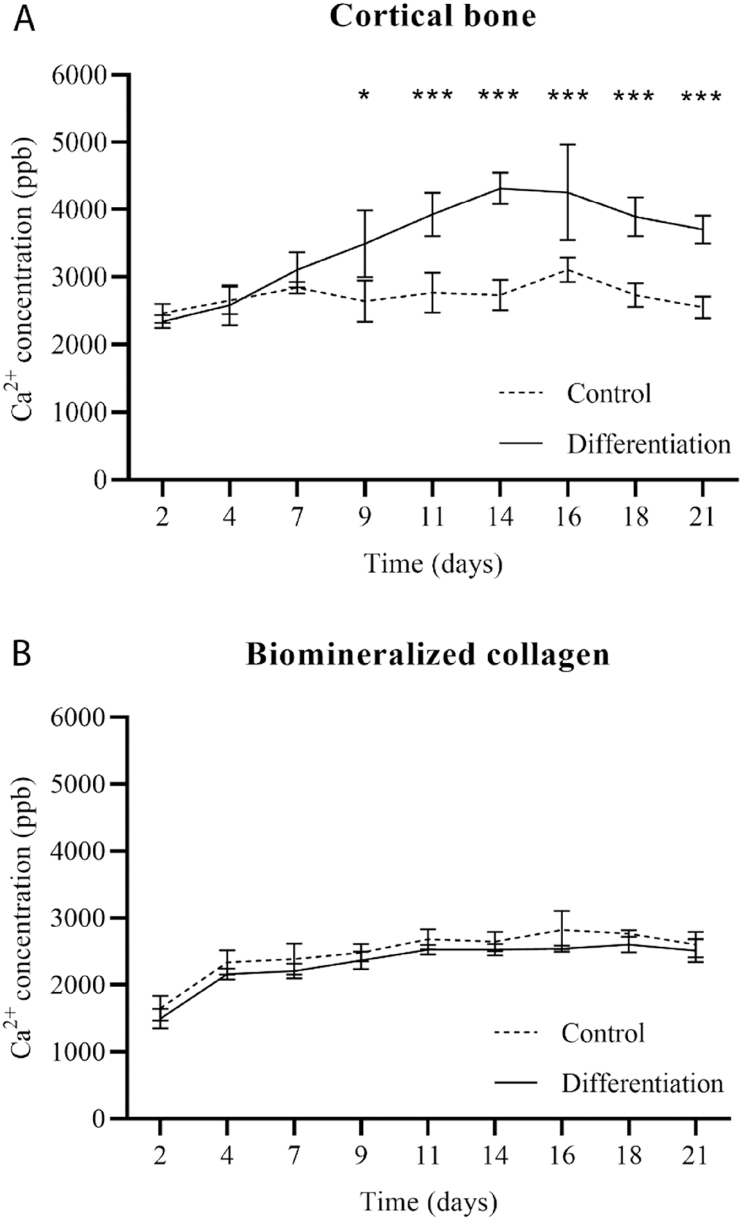
Concentration of calcium ions in cell culture medium, measured by ICP-MS at each moment of media change, for a total of 21 days. In the control condition cells were cultured in basic medium containing 20 ng/mL M-CSF, but without RANK-L, and in the differentiation condition cells were cultured in medium containing 20 ng/mL M-CSF and 40 ng/mL RANK-L. On cortical bone slices, differences between control and differentiation are visible from day 7 becoming significant from day 9 onwards. On biomineralized collagen surfaces, there are no appreciable differences in the calcium ion concentration between the control and differentiation conditions, indicating that no substantial (if any) resorption occurred.

To provide further evidence for resorption by osteoclasts, we measured the concentration of Ca^2+^ in cell culture medium over a 21-day experiment by using ICP-MS. The results showed that for cells cultured on cortical bone slices in differentiation medium, a higher Ca^2+^ concentration was observed between day 7 and day 21 in comparison with cells cultured in basic medium (Fig. 6A), with differences being statistically significant between day 9 and day 21. These results confirmed that there was ample resorption of cortical bone slices by the osteoclasts cultured on them over a period of 21 days. According to the profile of the Ca^2+^ concentration, it is suggested that the onset of osteoclast resorption occurred between day 4 and day 7, further increased up to day 16 and then leveled off until the end of the experiment at 21 days. It should be noted that the only difference between basic and differentiation medium is that in the latter, RANK-L was present. This means that effects such as CaP precipitation from cell culture medium (previously observed for CaP-containing materials [39]), or dissolution of the intrafibrillar mineral, are expected to occur in both basic and differentiation medium, and thus should not affect the differences between the two conditions.

In contrast to bone slices, the analysis of the cell culture medium of cells cultured on biomineralized collagen membrane in basic or differentiation medium showed no significant differences in Ca^2+^ concentration at any time point (Fig. 6B), suggesting that indeed, no measurable osteoclastic resorption occurred on this substrate, which is in line with other results.

### 3.4 Enzymatic activity of TRAP, CTS-K and CA-II does not necessarily correlate with osteoclast resorptive activity

To further characterize the osteoclasts that formed on different surfaces, and investigate to which extent the enzymatic activity of cells is predictive of osteoclast formation and resorptive activity, we performed enzyme activity assays on key osteoclast markers TRAP, CTS-K and CA-II after 7, 14 and 21 days of culture in basic or differentiation medium.

**Fig. 7:**
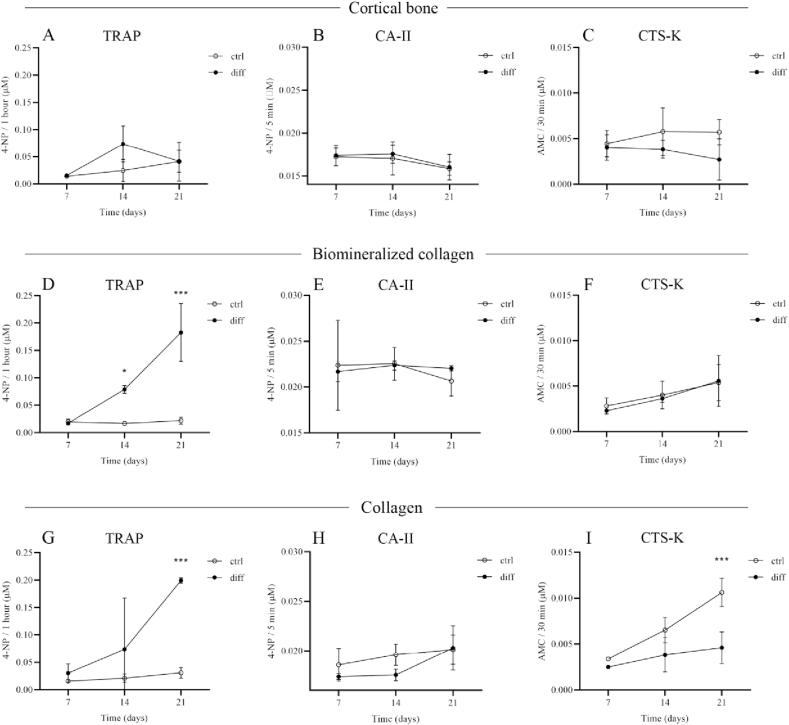
Activity of enzymes TRAP, CA-II and CTS-K, measured in lysates of osteoclasts cultured on cortical bone, biomineralized collagen, and collagen for 7, 14, and 21 days. TRAP activity was measured as the moles of 4-nitrophenyl converted during 1 h incubation with 4-nitrophenylphosphate. CA-II activity was measured as the moles of 4-nitrophenyl converted during 5 min incubation with 4-nitrophenylacetate. CTS-K activity was measured as the moles of aminomethylcoumarin converted during 30 min incubation with Z-LR-AMC.

During bone resorption, TRAP concentration in serum has been shown to increase, although its exact role in bone resorption is still not fully understood [40]. As is shown in Fig. 7A, at 7 days, the TRAP activity of cells cultured on bone slices was comparable between culture in basic and differentiation medium. Between 7 and 14 days, an increase in TRAP activity was observed in both basic and differentiation medium, however, this increase was more enhanced when cells were cultured in the differentiation medium. This resulted in higher activity in the differentiation medium than in the basic medium, although this difference was not statistically significant. Between 14 and 21 days, TRAP activity showed a further slight increase in basic medium, whereas a decrease was observed in differentiation medium, which resulted in comparable TRAP activity for the two conditions of cells cultured on bone slices for 21 days. The observed decrease in TRAP enzymatic activity between 14 and 21 days was in contrast with the immunofluorescence results (Fig. 2A and B), where TRAP activity nodules were still abundant after 21 days (Fig. 3A). The ICP-MS results (Fig. 6A) suggested that osteoclasts were actively resorbing up to day 21, although a decreasing trend in Ca^2+^ content of the medium between 14 and 21 days of culture was observed, which is suggestive of levelling off of resorptive activity and in line with a decrease in TRAP activity observed here.

For cells cultured on biomineralized collagen and collagen without mineral (Fig. 7D and 7G), there was an increase in TRAP activity between 7 and 21 days of culture in differentiation medium, whereas TRAP activity levels remained fairly constant when the culture was performed in basic medium. As a result, significantly higher TRAP activity values were measured in the differentiation medium after 14 and 21 days on both substrates, and they were comparable between collagen with and without mineral. These results confirmed the fluorescence data showing osteoclast formation and TRAP activity up to 21 days of culture in the differentiation medium on both substrates (Fig. 3B and 3C), although there was no proof of osteoclastic resorption. There is ample evidence that TRAP activity correlates well with osteoclastogenesis, with higher TRAP activity values indicating more osteoclasts, irrespective of the underlying substrate [[41], [42], [43]]. This evidence appears to extend to biomineralized collagen and collagen without mineral as well, as shown by the results of our study. However, correlation with resorption is less clear-cut, as it seems to depend on the substrate, but also on whether the enzyme is secreted or intracellular [25].

CA-II is an intracellular enzyme that acts as a proton source for acidification of the resorption lacunae [44]. Here, CA-II activity showed a different trend from TRAP activity on all surfaces. In the case of cortical bone slices (Fig. 7B), CA-II activity remained at the similar level between 7 and 14 days and decreased between 14 and 21 days for both cells cultured in basic and in differentiation medium. No differences were observed between the two media, at any time point. Similarly, for cells cultured on biomineralized collagen (Fig. 7E), no differences were observed between basic and differentiation medium at any of the time points. Moreover, no temporal changes were observed for either condition. Finally, on collagen matrices without mineral (Fig. 7H), in contrast to cortical bone and biomineralized collagen, a small increase in CA-II activity was observed between 7 and 21 days for both cells cultured in basic and in differentiation medium. At days 7 and 14, surprisingly, a higher CA-II activity was measured in basic than in differentiation medium. The results of CA-II activity do not seem to correlate with the TRAP activity results or the osteoclastogenesis observed by fluorescence immunohistochemistry. Such a correlation has been reported in literature [45], albeit on calcium phosphate cements, which osteoclasts were also shown to resorb. Our findings are in agreement with earlier work that the CA-II activity does not directly correlate with osteoclastic resorption [25].

Regarding the activity of CTS-K, a secreted protease responsible for type I collagen degradation during bone resorption [46], for cells cultured on cortical bone slices (Fig. 7C), no obvious temporal changes were observed, and a somewhat higher, though statistically not significant, CTS-K activity was found for cells cultured in basic than in differentiation medium after 14 and 21 days, which does not corroborate the pronounced resorptive activity in the differentiation medium as observed by immunofluorescence, SEM and ICP-MS. This might be because we measured enzyme activity in cell lysates, as opposed to extracellular, secreted CTS-K, which has previously been shown to correlate with resorbed area when cells were cultured on dentin slices [25]. On biomineralized collagen (Fig. 7F) and collagen without mineral (Fig. 7I), an increasing trend in CTS-K activity was observed between 7 and 21 days. While no differences were observed between cells cultured in basic and differentiation medium on biomineralized collagen, on collagen without mineral, higher CTS-K levels were measured in basic than in differentiation medium.

Taken together, these results indicate that intracellular enzymatic activity was not a reliable predictor of osteoclast resorption, as there was little correlation among TRAP, CA-II and CTS-K activity in cell lysates. Moreover, the activity of these enzymes did not reflect the evidence for osteoclast formation and resorptive activity as obtained using immunohistochemistry and morphological investigation using SEM. Other factors, such as location of enzyme (intra- *versus* extracellular), composition of the biomaterial, and pH of the culture medium may have all played a role in these results. For quantification of resorption, ICP-MS analysis of Ca^2+^ concentration in cell culture medium proved to be a more useful indicator of resorptive activity on calcium-containing biomaterials.

The results presented here show that while osteoclast-like cell formation seems to readily occur on biomineralized collagen, substrate resorption does not. No clearly defined actin rings were observed on biomineralized or pure collagen, and no resorption of these materials was detected. We hypothesize that the two main factors contributing to this are i) the porous, mesh-like structure of the membranes; and ii) the lack of ligands to the vitronectin receptor. The large pores of the mesh-like fibrillary collagen are likely to have prevented the normal formation of actin rings, a structure that is key to sealing off a section of material for degradation, and without which no resorption can take place. Moreover, the lack of ligands to the vitronectin receptor may have further contributed to this effect.

Despite its biomimetic design, biomineralized collagen, as prepared, was not resorbed like cortical bone. Although the biomineralized collagen showed closer-to-native properties than other materials to date, it is clear that, as far as osteoclast function is concerned, a key aspect of the native tissue was not replicated sufficiently. We suggest that a more dense substrate, with pore size reduced to the submicron scale, would likely support osteoclast resorption. Therefore the next steps in developing biomineralized collagen materials should focus on densification of the matrix, with either closer packing of the collagen fibers, or with extrafibrillar mineral to reduce porosity. Moreover, functionalizing the material with appropriate ligands to the vitronectin receptor is expected to enhance its ability to be resorbed.

## 4 Conclusion

We investigated osteoclast formation and activity on biomineralized collagen and collagen membranes and made a comparison with cortical bone, a material that is well known for supporting normal osteoclast resorption. We found that biomineralized collagen and collagen membranes support osteoclast-like cell formation via RANK-L induced fusion of monocyte/macrophage precursors. Osteoclasts expressed TRAP on all materials, but only formed clear actin rings on cortical bone, which was also the only material osteoclasts were able to resorb. We hypothesized that the fibrillar, porous structure of the collagen membrane's surface, together with the lack of RGD ligands to the vitronectin receptor, prevented the formation of a stable sealing zone. Toward the development of more biomimetic biomineralized collagen composites, future studies should focus on the role of material porosity and fibrillary structure in directing osteoclast actin ring formation and resorption. Progress in these areas may result in better bone graft substitutes that can be more readily remodeled by osteoclasts and substituted by new bone tissue.

## Data availability statement

The raw data generated for this study are publicly available at https://doi.org/10.34894/UAL6GN.

## Author contributions

DP designed and performed the experiments. ND and PH provided critical input in experimental design. DP, ND and PH wrote the manuscript. PH reviewed the manuscript. PH was responsible for funding acquisition, project administration and supervision. All authors contributed to the article and approved the submitted version.

## Funding

This research has been made possible with the support of the Dutch Province of Limburg (LINK project). PH gratefully acknowledges the Gravitation Program ‘Materials-Driven Regeneration’, funded by the 10.13039/501100003246Netherlands Organisation for Scientific Research (NWO).

## Declaration of competing interest

The authors confirm that there are no known conflicts of interest associated with this publication and there has been no significant financial support for this work that could have influenced its outcome.
