# Supplementary material for: Human osteoclast formation and resorptive function on biomineralized collagen
Source: Bioact Mater. 2021 Jul 15;8:241–52. doi: 10.1016/j.bioactmat.2021.06.036 (PMC8424427; doi:10.1016/j.bioactmat.2021.06.036)
Supplement: Multimedia component 1 [file mmc1.docx]

**Supplementary Material**


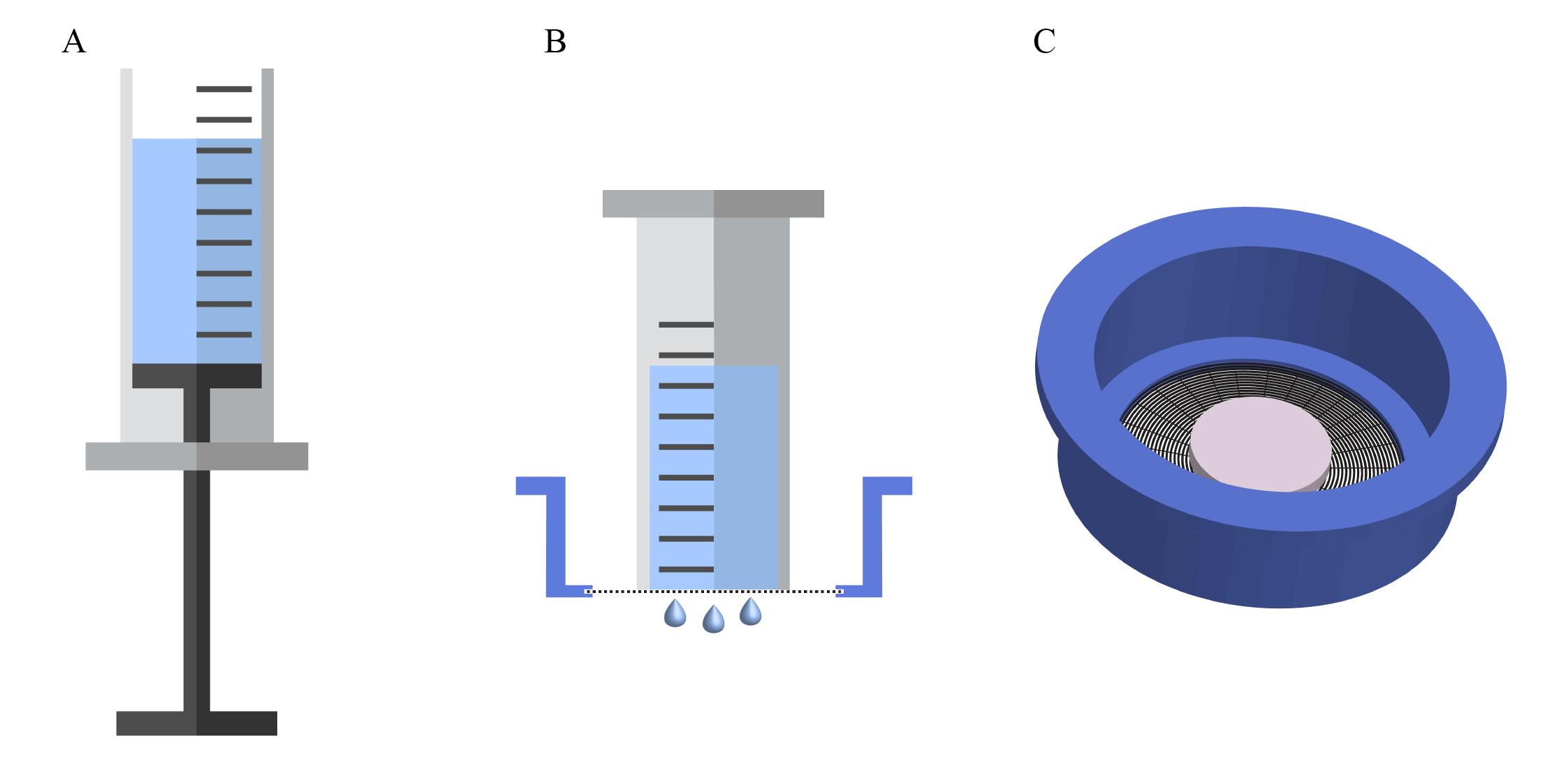


Supplementary Figure 1. Method for preparation of collagen membranes. Gel formation in upright modified syringe for 24 hours (A); piston removal and syringe body placement on a cell strainer with a nylon mesh of 40 µm pore size for 24 hours (B); removal of syringe body, flattened collagen membrane remains on the cell strainer for another 24 hours (C).
